# Biventricular takotsubo syndrome complicated with cardiogenic shock and shark fin sign requiring ECPELLA: a case report

**DOI:** 10.1093/ehjcr/ytaf366

**Published:** 2025-07-28

**Authors:** Kanta Takeda, Ryohei Ono, Ken Kato, Togo Iwahana, Yoshio Kobayashi

**Affiliations:** Department of Cardiovascular Medicine, Chiba University Graduate School of Medicine, 1-8-1 Inohana, Chuo-ku, Chiba 260-8670, Japan; Department of Cardiovascular Medicine, Chiba University Graduate School of Medicine, 1-8-1 Inohana, Chuo-ku, Chiba 260-8670, Japan; Department of Cardiovascular Medicine, Chiba University Graduate School of Medicine, 1-8-1 Inohana, Chuo-ku, Chiba 260-8670, Japan; Department of Cardiovascular Medicine, Chiba University Graduate School of Medicine, 1-8-1 Inohana, Chuo-ku, Chiba 260-8670, Japan; Department of Cardiovascular Medicine, Chiba University Graduate School of Medicine, 1-8-1 Inohana, Chuo-ku, Chiba 260-8670, Japan

**Keywords:** Biventricular failure, Cardiogenic shock, ECMO, Impella, Mechanical circulatory support, Shark fin sign, Takotsubo syndrome

## Abstract

**Background:**

Takotsubo syndrome (TTS) is a transient cardiac condition primarily affecting left ventricular function and is often triggered by physical or emotional stress. Biventricular involvement in TTS has been recently reported, and such cases are associated with a more severe clinical presentation. However, biventricular TTS with cardiogenic shock (CS) requiring mechanical circulatory support (MCS) is rare. Furthermore, shark fin sign (SFS) is a distinctive electrocardiographic pattern typically associated with significant myocardial ischaemia, but SFS associated with TTS has seldom been reported.

**Case summary:**

A 77-year-old woman with sepsis and pyelonephritis presented with fever and chest pain. An initial electrocardiogram showed SFS. Transthoracic echocardiography revealed a severely reduced left ventricular ejection fraction with apical ballooning, akinesis of the apical free-wall segment, and hyperkinesia of the basal segments of the right ventricle. Impella^®^ was inserted for CS, but the shock persisted due to severe right ventricular (RV) dysfunction, and she required veno-arterial extracorporeal membrane oxygenation. After the treatments, her cardiac functions improved, and MCS was weaned off. The follow-up findings of electrocardiographic changes and Thallium-201 and iodine-123-metaiodobenzylguanidine (MIBG) myocardial scintigraphy were consistent with TTS.

**Discussion:**

In the case of biventricular TTS with CS, the use of MCS for RV support may be required. Shark fin sign may be associated with haemodynamic instability or shock even in TTS. Furthermore, repeated myocardial scintigraphy is useful for diagnosing TTS because the acute phase shows preserved myocardial perfusion with markedly reduced MIBG uptake indicating impaired sympathetic innervation, whereas the chronic phase shows improvement in MIBG uptake.

Learning points

- Biventricular takotsubo syndrome (TTS) is rare but may present with more severe clinical manifestations compared to the typical TTS, which involves only the left ventricle.
- Severe haemodynamic instability is more frequently observed in biventricular TTS, sometimes necessitating more intensive management strategies, including mechanical circulatory support.
- Shark fin sign generally represents a large burden of myocardial ischaemia, but this sign can also be seen in TTS.

## Introduction

Takotsubo syndrome (TTS), also known as stress-induced cardiomyopathy, is a transient cardiac condition typically involving reversible left ventricular (LV) dysfunction in the absence of obstructive coronary artery disease.^1^ It is most often triggered by emotional or physical stress.^1^ The classic form presents with apical ballooning, although mid-ventricular and basal variants have also been described. While TTS usually affects only the left ventricle, some cases with biventricular involvement have recently been reported.^2,3^ Among these, biventricular TTS with cardiogenic shock (CS) requiring mechanical circulatory support (MCS) is rare. In addition, the shark fin sign (SFS) is a distinctive electrocardiographic pattern typically associated with significant myocardial ischaemia, but TTS with SFS have rarely been reported.^4,5^ Herein, we report a unique case of biventricular TTS complicated with CS and SFS on electrocardiogram requiring ECPELLA, which involves the concomitant use of extracorporeal membrane oxygenation (ECMO) and percutaneous ventricular assist device (Impella^®^).

## Summary figure

**Table:** 

Day 0	The patient with sepsis and pyelonephritis presented with chest pain. An electrocardiogram revealed a shark fin sign, and echocardiography showed biventricular systolic dysfunction with basal hyperkinesia, consistent with biventricular takotsubo syndrome. Mechanical circulatory support with ECPELLA was initiated.
Day 4	ECMO was weaned off.
Day 8	Impella^®^ was explanted.
Day 12	Extubation was performed.
Day 22	Thallium-201 (Tl) and iodine-123-metaiodobenzylguanidine (MIBG) myocardial scintigraphy showed a mismatch between Tl and MIBG uptake, supporting the diagnosis of TTS.
Day 24	Cardiac magnetic resonance imaging showed high signals at the mid-to-apical myocardium of the left ventricle on T2 weighted imaging and mid wall late gadolinium enhancement in the interventricular septum.
Day 35	The patient was discharged.
3 months	Follow-up echocardiography and Tl/MIBG myocardial scintigraphy showed improvement of biventricular functions.

## Case presentation

A 77-year-old woman with a history of renal oncocytoma, angina pectoris, hypertension, and diabetes mellitus was referred to our hospital after experiencing chest pain and dyspnoea on Day 3 of hospitalization for sepsis. Her past medical history included stable angina, for which she had undergone percutaneous coronary intervention (PCI) to the left anterior descending artery 1 year earlier. Three months prior, she underwent robot-assisted partial nephrectomy for a left renal tumour, which was pathologically diagnosed as renal oncocytoma. She was a past smoker (20 cigarettes/day for 15 years), and her family history was unremarkable.

**Figure 1:**
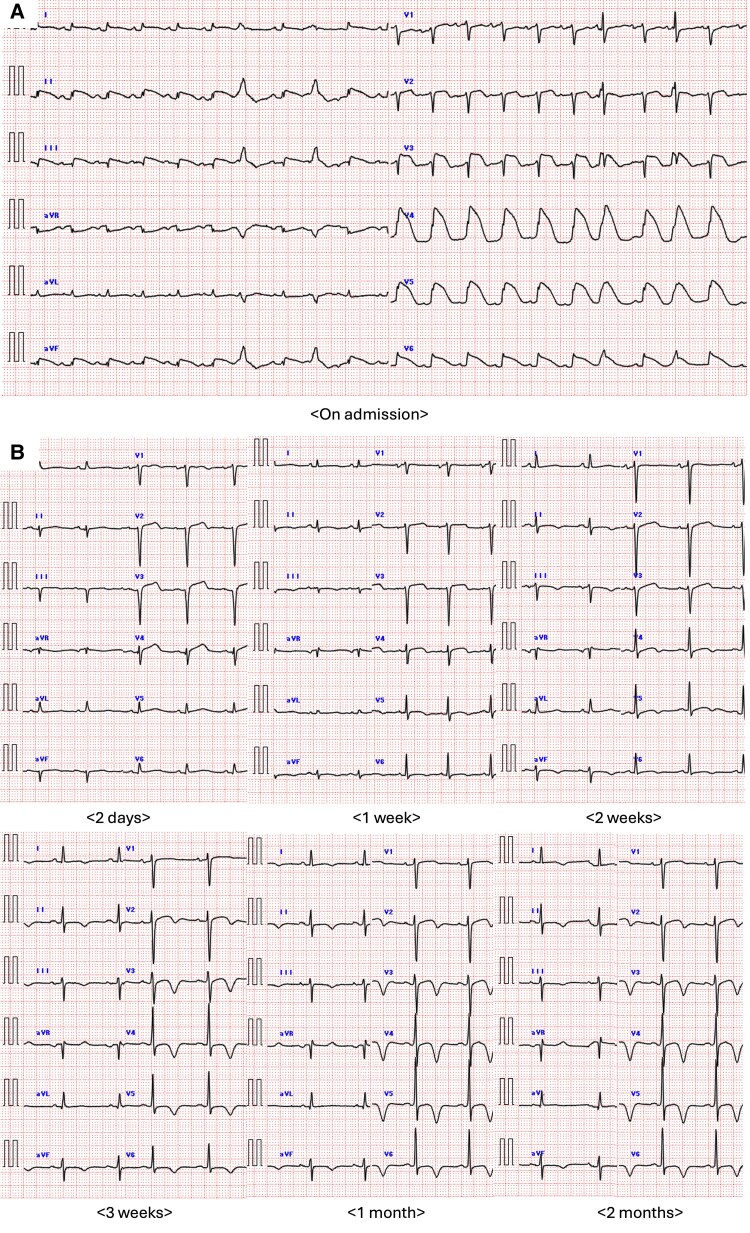
(*A*) Electrocardiogram on admission. (*B*) Electrocardiogram changes during 2-month follow-up.

**Figure 2:**
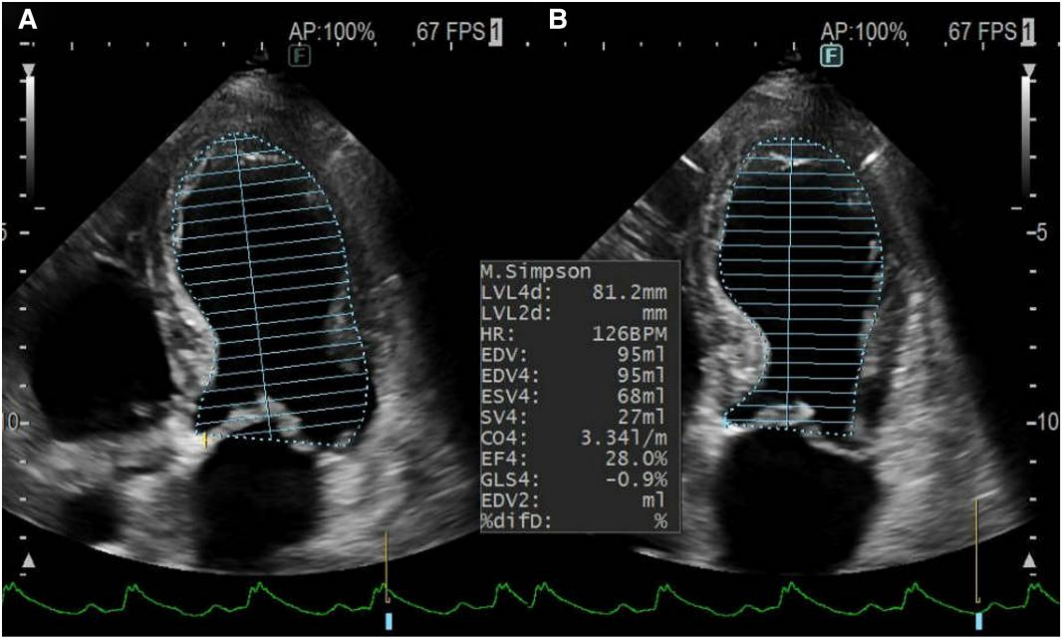
Transthoracic echocardiography demonstrating severely reduced left ventricular ejection fraction (28%, Simpson) with mid-to-apical hypokinesia and apical ballooning. (*A*) Diastolic phase and (*B*) systolic phase.

**Figure 3:**
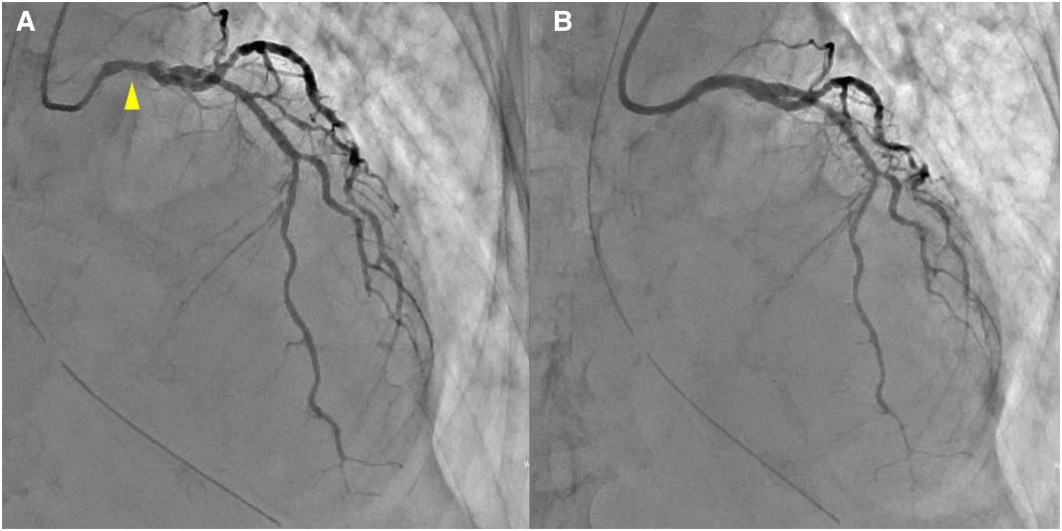
(*A*) Coronary angiography showing moderate stenoses of the left main stem (arrowhead) and left anterior descending artery. (*B*) Coronary angiography after percutaneous coronary intervention.

Two days prior to referral, she experienced fever and hypotension, and was diagnosed with sepsis and pyelonephritis at another hospital. She was started on intravenous ceftriaxone (2 g/day) and admitted. Blood and urine cultures revealed growth of *Escherichia coli*, which was sensitive to ceftriaxone. However, due to the development of chest pain and hypoxia, she was referred and transferred to our hospital for further management. Her vital signs were as follows: blood pressure (BP), 120/70 mmHg; heart rate, 130 beats/min; oxygen saturation, 96% at 12 L O_2_/min; and body temperature, 37.6°C. Physical examination revealed coarse crackles on lung auscultation, cold extremities, and no peripheral oedema. An electrocardiogram showed tachycardia and ST-T segment elevations in leads I, II, III, aVF, and V2–6; of note, shark fin signs were noted in leads II, III, aVF, and V4–6 (*Figure 1A*). Chest X-ray revealed significant pulmonary congestion. Transthoracic echocardiography demonstrated severely reduced left ventricular ejection fraction (LVEF, 28%) with apical ballooning (*Figure 2*). Additionally, akinesis of the apical free-wall segment and hyperkinesia of the basal segments of the right ventricle were noted (see Supplementary material online, *Video S1*). Laboratory data on the day of transfer and presentation to the emergency department at our hospital showed elevation of white blood cells, C-reactive protein, creatine kinase (CK), CK-myocardial band (CK-MB), troponin I, and brain natriuretic peptide (see Supplementary material online, *Table S1*). Due to severe dyspnoea, she was intubated and underwent emergent coronary angiography, which revealed moderate stenoses of the left main stem and left anterior descending artery (*Figure 3A*). As ischaemia could not be ruled out (see Supplementary material online, *Videos S2* and *S3*), PCI was subsequently performed (*Figure 3B*). Although no acute lethal arrhythmia, tamponade, or coronary perforation was present, her BP continued to decline, and Impella CP^®^ was inserted for haemodynamic support. Soon after starting Impella CP^®^, her BP dropped further due to right-sided heart failure, and veno-arterial ECMO (VA-ECMO) was added. Her peak CK and CK-MB levels were those at admission; given the clinical course of biventricular heart failure requiring VA-ECMO followed by Impella CP^®^ and no remarkable elevation of CK enzymes, biventricular TTS was highly suspected.

**Figure 4:**
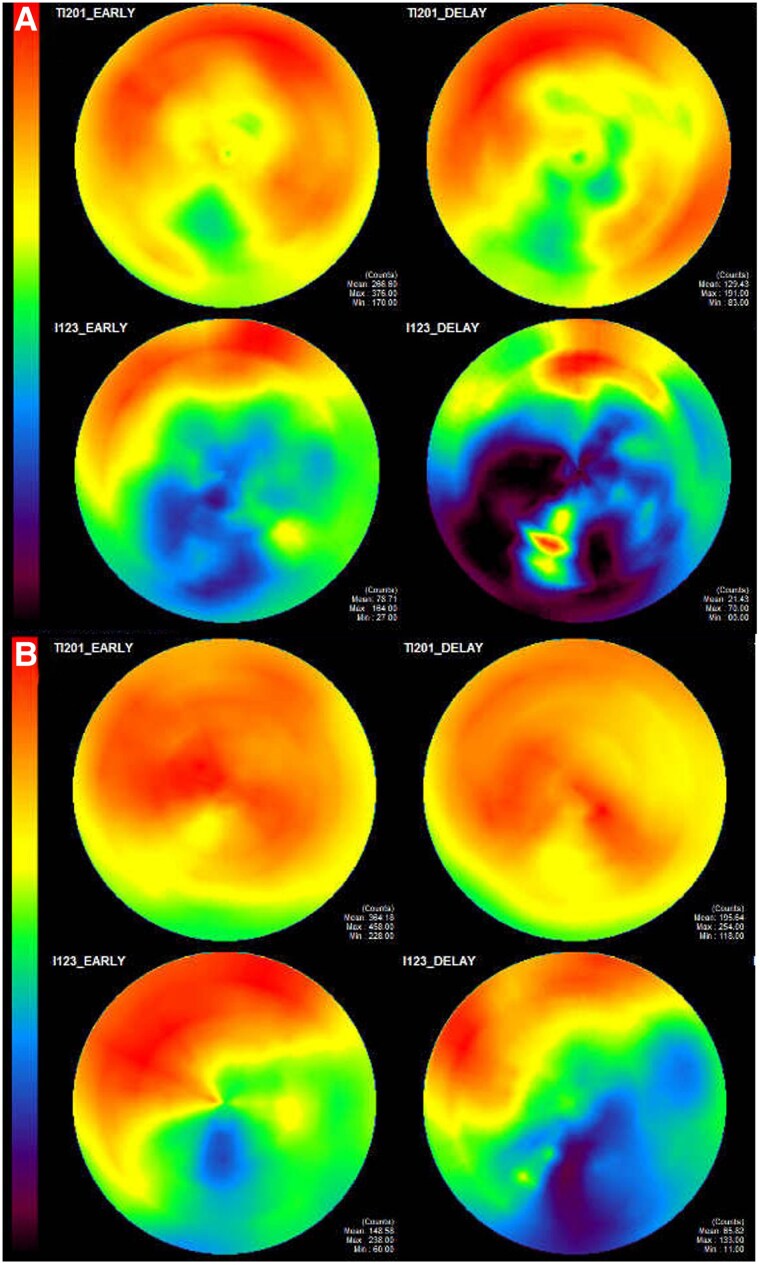
Thallium-201 (Tl) and iodine-123-metaiodobenzylguanidine myocardial scintigraphy. (*A*) Tl scintigraphy on Day 22 showing decreased uptake in the inferior wall and iodine-123-metaiodobenzylguanidine scintigraphy showing a marked reduction in uptake across a wide area other than the anterior wall and septum areas. (*B*) Tl and iodine-123-metaiodobenzylguanidine myocardial scintigraphy two months after the discharge showing improvement of the iodine-123-metaiodobenzylguanidine uptakes in the left ventricle.

She was initially treated with MCS, dobutamine, and norepinephrine for acute decompensated heart failure. Her cardiac functions via imaging gradually improved and VA-ECMO and Impella CP^®^ were successfully weaned off on Days 4 and 8, respectively, and the patient was extubated on Day 12. Follow-up blood and urine cultures were negative and the antibiotic was discontinued on Day 14. On Day 21, transthoracic echocardiography was performed to assess recovery from acute haemodynamic instability, evaluate the course of suspected TTS, and monitor early response to optimal medical therapy, showing an improvement in LVEF to 36%, although apical akinesis persisted. Regarding the medications, bisoprolol was initiated on the day after MCS withdrawal (Day 9 of admission) and its dosage was gradually increased to 5 mg/day. Additionally, spironolactone 25 mg/day was initiated on Day 18, followed by sacubitril/valsartan 24 mg/26 mg twice a day on Day 23. Sodium-glucose cotransporter-2 inhibitor was not administered because of urosepsis. Thallium-201 (Tl) and iodine-123-metaiodobenzylguanidine (MIBG) myocardial scintigraphy was performed on Day 22. Thallium-201 scintigraphy showed decreased uptake in the inferior wall; in contrast, MIBG scintigraphy demonstrated a marked reduction in uptake across a wide area other than the anterior wall and septum areas (*Figure 4A*). This finding indicates a mismatch between Tl and MIBG uptake, suggesting TTS. Furthermore, cardiac magnetic resonance imaging (CMR) on Day 24 showed high signals at the mid-to-apical myocardium of the LV in T2 weighted images. A quantitative analysis of T1 mapping of the myocardium at the apex showed a marked native T1 prolongation of 1500 ms. Late gadolinium enhancement was mainly observed in the middle layer of the LV septum (see Supplementary material online, *Figure S1*). These findings were unlikely to indicate ischaemia or myocarditis. She was discharged on Day 35 without any complication.

Electrocardiogram changes showed that the negative T-waves in leads I, II, III, aVF, and the precordial leads gradually deepened over the course of two months (*Figure 1B*). Follow-up transthoracic echocardiography 2 months after the discharge was performed to evaluate the response to optimal medical therapy and showed improvement of biventricular systolic functions (see Supplementary material online, *Video S4*), and T1 and MIBG myocardial scintigraphy on the same day also demonstrated improvement of the MIBG uptakes in the LV (*Figure 4B*). At the follow-up, the patient reported no symptoms and had returned to her normal daily activities without limitations.

## Discussion

This case is remarkable for three reasons. Firstly, patients diagnosed with biventricular TTS requiring MCS have rarely been reported.^6^ Secondly, shark fin sign is not so common in TTS. Finally, the detailed follow-up was helpful in understanding the progression of this unique pathology.

Regarding the first point, the patient experienced CS due to biventricular involvements. To diagnose biventricular TTS, echocardiography or CMR is often used.^7^ In general, CS due to TTS has been reported with an incidence ranging from 1% to 20%.^7^ In the presence of right ventricular (RV) involvement of TTS with CS, physicians should be careful when using Impella^®^ alone, because inadequate support of RV function would result in circulatory failure from the right ventricle to the LV, leading to haemodynamic instability.^7,8^ Regarding selection of the MCS, intra-aortic balloon pumping was considered unlikely to function effectively due to tachycardia in our case. Given the patient's shock state and severely impaired LV function, Impella^®^ was placed initially, but the patient’s BP dropped and ECMO was required. Therefore, biventricular TTS with CS may require RV support with ECPELLA or biventricular Impella^®^.

Regarding the second point, SFS is an electrocardiogram pattern with (i) a giant R wave (amplitude ≥1 mV) and (ii) a QRS complex fused with the ST-segment and the T-wave.^5^ Shark fin sign is generally associated with a large burden of myocardial ischaemia, but a few cases of shark fin sign associated with TTS have been reported, and we reviewed the literature (*Table 1*).^4^

**Table 1:** Literature review of shark fin sign in patients with takotsubo syndrome

Author	Age	Sex	Comorbidities	Leads presenting ‘shark fin sign’ morphology	LVEF (%)	sepsis	Vasopressors	Mechanical ventilation	Mechanical circulatory support	Prognosis
Almutairi	57	F	Colorectal carcinoma, adhesive intestinal obstruction, bowel perforation	I, aVL, V2–6	<25	No	Yes	Yes	No	Alive
Joki	67	F	None	II, III, aVF, V3–6	20	No	Yes	No	No	Alive
Verdoia	51	F	Epilepsy, HT, DM, personality disorder, alcohol abuse, pyometra, extraneous body	I, aVL, V2–6	30	Yes	Yes	Yes	No	Alive
Zhang	54	F	ND	II, III, aVF, V3–6	38	Yes	Yes	No	No	Alive
Takeda (our case)	77	F	Renal oncocytoma, angina pectoris, HT, and DM	II, III, aVF, V4–6	28	Yes	Yes	Yes	Yes (ECPELLA)	Alive

DM, diabetes mellitus; ECPELLA, extracorporeal membrane oxygenation and Impella^®^; F, female; HT, hypertension; LVEF, left ventricular ejection fraction; ND, not described.

All cases were aged above 50, female, and with LVEF < 40%. Three of the five cases had sepsis; thus, such physical stressful events may be associated with the onset of TTS. All cases received vasopressor treatment, and mechanical ventilation was required in three of the five cases; however, only our case required ECPELLA. All cases survived.

Regarding the last point, we performed repeated myocardial scintigraphy, and our findings were consistent with TTS. In Tl and MIBG scintigraphy for TTS, the acute phase shows preserved myocardial perfusion with markedly reduced MIBG uptake indicating impaired sympathetic innervation, whereas the chronic phase shows improvement in MIBG uptake.^9,10^ Therefore, myocardial scintigraphy is very useful for differentiating TTS from ischaemic heart disease.

In conclusion, we report a unique case of biventricular TTS complicated with CS requiring ECPELLA and associated with SFS on electrocardiogram. Biventricular TTS is rare but may present with more severe clinical manifestations compared to the typical TTS, and physicians should be aware that RV support with MCS may be required when treating biventricular TTS with CS.

## Supplementary Material

ytaf366_Supplementary_Data

## Data Availability

The data underlying this article will be shared on reasonable request to the corresponding author.

## Lead author biography

Mr Kanta Takeda is a medical student at Chiba University School of Medicine. He is interested in general cardiology.

## References

[1] Ono R, Falcao LM. Takotsubo cardiomyopathy systematic review: pathophysiologic process, clinical presentation and diagnostic approach to Takotsubo cardiomyopathy. Int J Cardiol 2016;209:196–205. 26896623 10.1016/j.ijcard.2016.02.012

[2] El-Battrawy I, Santoro F, Stiermaier T, Möller C, Guastafierro F, Novo G, et al Incidence and clinical impact of right ventricular involvement (biventricular ballooning) in Takotsubo syndrome: results from the GEIST registry. Chest 2021;160:1433–1441. 34052189 10.1016/j.chest.2021.04.072

[3] Kato K, Ishibashi I, Ghadri JR, Templin C. Biventricular takotsubo syndrome. Eur Heart J 2019;40:2171. 30977799 10.1093/eurheartj/ehz198

[4] Ohnaga Y, Ono R, Aoki K, Kato H, Iwahana T, Takaoka H, et al Shark fin sign in a patient with sepsis-induced cardiomyopathy associated with retained placenta: a case report and review of the literature. Intern Med 2024;64:1047–1052. 39231682 10.2169/internalmedicine.3501-24 PMC12021502

[5] Ono R, Iwahana T, Kato H, Aoki K, Kobayashi Y. Shark fin sign. Am J Med 2024;137:e35–e37. 37875220 10.1016/j.amjmed.2023.10.004

[6] Mierke J, Loehn T, Linke A, Ibrahim K. Reverse takotsubo cardiomyopathy-life-threatening symptom of an incidental pheochromocytoma: a case report. Eur Heart J Case Rep 2019;3:1–6. 10.1093/ehjcr/ytz195 PMC7026602 32099962

[7] Kato K, Di Vece D, Kitagawa M, Yamamoto K, Aoki S, Goto H, et al Cardiogenic shock in takotsubo syndrome: etiology and treatment. Cardiovasc Interv Ther 2024;39:421–427. 39039401 10.1007/s12928-024-01031-3 PMC11436465

[8] Citro R, Bossone E. Negative prognostic impact of biventricular ballooning in takotsubo syndrome: when two is not better than one. Chest 2021;160:1179–1180. 34625168 10.1016/j.chest.2021.06.013

[9] Akashi YJ, Nakazawa K, Sakakibara M, Miyake F, Musha H, Sasaka K. 123I-MIBG myocardial scintigraphy in patients with “takotsubo” cardiomyopathy. J Nucl Med 2004;45:1121–1127. 15235057

[10] Akashi YJ, Takano M, Miyake F. Scintigraphic imaging in takotsubo cardiomyopathy. Herz 2010;35:231–238. 22086475 10.1007/s00059-011-3445-4

