# Supplementary material for: Biventricular takotsubo syndrome complicated with cardiogenic shock and shark fin sign requiring ECPELLA: a case report
Source: Eur Heart J Case Rep. 2025 Jul 28;9(8):ytaf366. doi: 10.1093/ehjcr/ytaf366 (PMC12342952; doi:10.1093/ehjcr/ytaf366)
Supplement: ytaf366_Supplementary_Data [file ytaf366_supplementary_data.zip › Table S1.docx]

**Table S1.** Laboratory data on the day of transfer and presentation to the emergency department at our hospital.

WBC, white blood cells; Hb, hemoglobin; Plt, platelets; TP, total protein; Alb, albumin; T-Bil, total bilirubin; D-Bil, direct bilirubin; AST, aspartate aminotransferase; ALT, alanine transaminase; LDH, lactate dehydrogenase; γGTP, γ-glutamyl transpeptidase; ALP, alkaline phosphatase; CK, creatine kinase; CK-MB, creatine kinase-myocardial band; TnI, Troponin I; BUN, blood urea nitrogen; Cre, Creatinine; eGFR, estimated glomerular filtration rate; CRP, C reactive protein; FBS, fasting blood sugar; HbA1c, hemoglobin A1C; BNP, brain natriuretic peptide precursor.

|  |  |  | Reference |
| --- | --- | --- | --- |
| WBC | 15200 | /μL | (3300-8600) |
| Hb | 11.3 | g/dL | (11.6-14.8) |
| Plt | 16.7 | 10^4^/μL | (15.8-34.8) |
| TP | 6.8 | g/dL | (6.6-8.1) |
| ALB | 3.4 | g/dL | (4.1-5.1) |
| T-Bil | 0.5 | mg/dL | (0.4-1.5) |
| D-Bil | 0.1 | mg/dL | (<0.2) |
| AST | 185 | U/L | (13-30) |
| ALT | 46 | U/L | (7-23) |
| LDH | 549 | U/L | (124-222) |
| γGTP | 21 | U/L | (9-32) |
| ALP | 59 | U/L | (38-113) |
| CK | 853 | U/L | (41-153) |
| CK-MB | 37.9 | ng/mL | (<3.1) |
| TnI | 32639 | pg/mL | (<15.6) |
| BUN | 33 | mg/dL | (8-20) |
| Cre | 1.29 | mg/dL | (0.46-0.79) |
| eGFR | 31.2 | mL/min/1.73m² | |
| Na | 130 | mEq/L | (138-145) |
| K | 3.8 | mEq/L | (3.6-4.8) |
| Cl | 97 | mEq/L | (101-108) |
| CRP | 21.69 | mg/dL | (<0.14) |
| FBS | 289 | mg/dL | (73-109) |
| HbA1c | 6.1 | % | (4.9-6.2) |
| BNP | 39282 | pg/mL | (<18.4) |
