# Supplementary figures and images for: Biventricular takotsubo syndrome complicated with cardiogenic shock and shark fin sign requiring ECPELLA: a case report
Source: Eur Heart J Case Rep. 2025 Jul 28;9(8):ytaf366. doi: 10.1093/ehjcr/ytaf366 (PMC12342952; doi:10.1093/ehjcr/ytaf366)

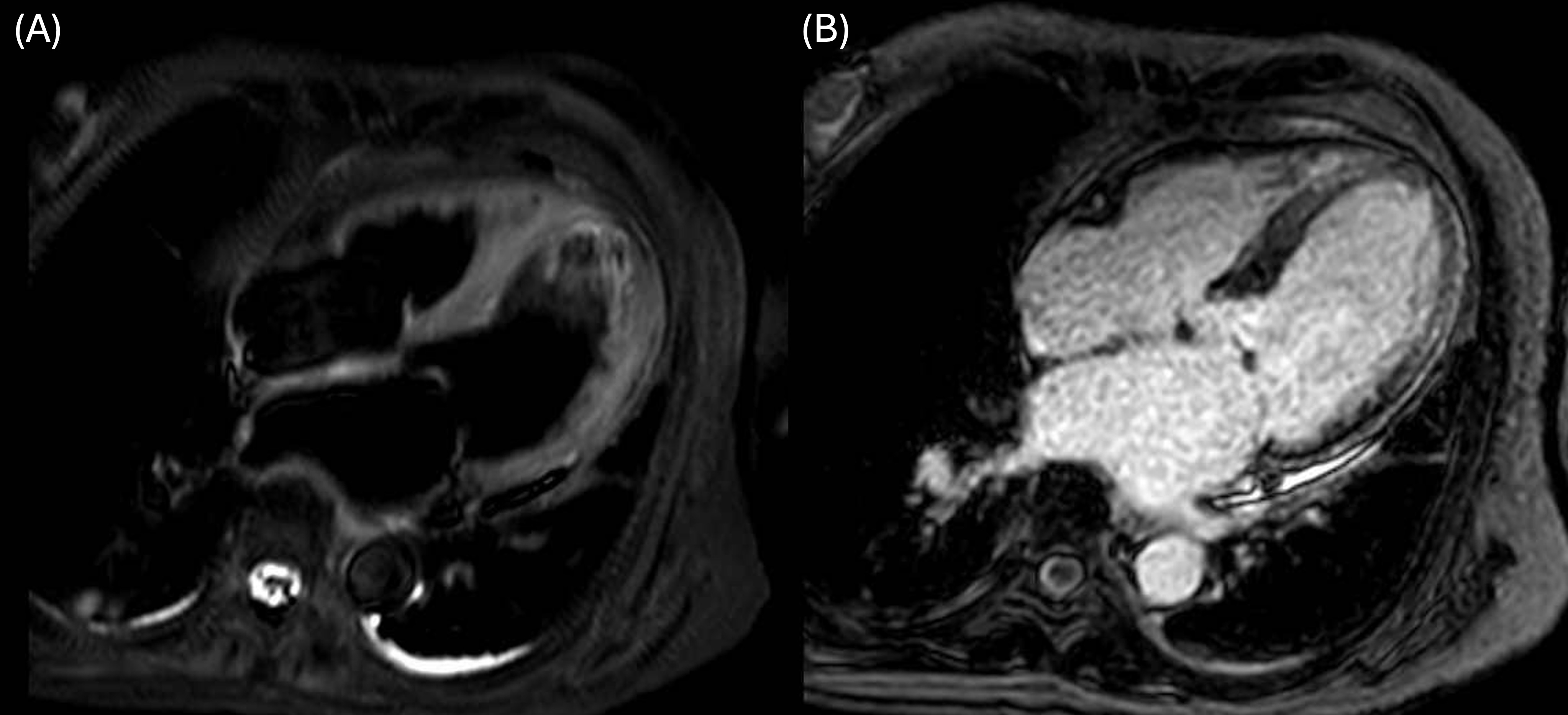

Supplement: ytaf366_Supplementary_Data [file ytaf366_supplementary_data.zip › Suppl Fig 1.jpg]
